# Synergistic antimalarial treatment of *Plasmodium berghei* infection in mice with dihydroartemisinin and *Gymnema inodorum* leaf extract

**DOI:** 10.1186/s12906-023-03850-y

**Published:** 2023-01-23

**Authors:** Sakaewan Ounjaijean, Voravuth Somsak

**Affiliations:** 1 Research Institute for Health Sciences, Chiang Mai University, 50200 Chiang Mai, Thailand; 2 Environmental-Occupational Health Sciences and Non-Communicable Diseases Research Group (EOHS and NCD Research Group), Research Institute for Health Sciences, Chiang Mai University, 50200 Chiang Mai, Thailand; 3 School of Allied Health Sciences, Walailak University, 80160 Nakhon Si Thammarat, Thailand; 4 Research Excellence Center for Innovation and Health Products, Walailak University, 80160 Nakhon Si Thammarat, Thailand

**Keywords:** Antimalarials, Dihydroartemisinins, *Gymnema inodorum*, *Plasmodium berghei*

## Abstract

**Background:**

Chemotherapy is crucial in the fight against malaria. The rise of resistance to most antimalarial medicines has been a serious hurdle to effective treatment. Artemisinin-based combination therapies (ACTs) are currently the most effective antimalarial medication. Malaria parasites are growing more resistant to ACTs, particularly in Southeast Asia. As a result, effective alternative antimalarials are in high demand. The leaf extract of *Gymnema inodorum* (GIE) has previously shown promise as an effective antimalarial. Therefore, this study evaluated the antimalarial potential of combination dihydroartemisinin (DHA) and GIE therapy against *Plasmodium berghei* in a mouse model.

**Methods:**

The medications were evaluated using the standard 4-day test for determining the 50% effective dosage (ED_50_) of DHA and GIE on *P. berghei* ANKA (PbANKA). DHA and GIE were combined using a fixed-ratio approach, with DHA/GIE ED_50s_ of 100/0, 80/20, 60/40, 40/60, 20/80, and 0/100, respectively.

**Results:**

The ED_50_ against PbANKA was determined to be 2 mg/kg of DHA and 100 mg/kg of GIE. The 60/40 (DHA/GIE) ratio demonstrated significantly higher antimalarial activity than the other ratios (*p* < 0.001) against PbANKA, with 88.95% inhibition, suggesting synergistic efficacy (combination index (CI) = 0.68695). Furthermore, this ratio protected PbANKA-infected mice against loss of body weight and packed cell volume decline, leading to a longer survival time over 30 days.

**Conclusion:**

Our results suggest that GIE could be an effective adjuvant to DHA that can enhance the antimalarial effects in the treatment of PbANKA-infected mice.

## Background

Malaria is a parasitic disease that mainly affects people in developing countries in Africa, Asia, Latin America, and the South-West Pacific. *Plasmodium falciparum*, *Plasmodium vivax*, *Plasmodium malariae*, *Plasmodium ovale*, *and Plasmodium knowlesi* are Apicomplexan protozoan parasites that cause malaria in humans and are spread by the female *Anopheles* mosquito [1]. Since 2000, approximately 1.5 billion malaria cases have been avoided, with 229 million new cases and 409,000 fatalities reported in 2019, primarily among pregnant women and children under the age of five [2]. Despite problems with the development of effective vaccines and vector control, chemotherapy remains the foundation of malaria management strategies. However, the emergence and spread of malaria parasites resistant to many of today’s antimalarial medications is a major source of concern [3]. Artemisinin-based combination therapy (ACT) was developed to address this and is now recommended by the World Health Organization as the first-line treatment for uncomplicated malaria [4]. Unfortunately, artemisinin resistance, reducing the efficacy of ACT, has been reported along the Thailand-Cambodia border in Southeast Asia [5]. As a result, there is still a pressing need to identify and develop new malaria treatments, and combinations that incorporate medicinal plants are among the potential sources of such treatments. Traditional medicine has long been used to treat a wide range of medical conditions, including malaria. Many secondary metabolites produced by medicinal plants are now understood to contribute to a variety of therapeutic actions. The WHO recognizes the value of traditional medicines and continues to advocate their inclusion in national health systems [6].

*Gymnema inodorum (Lour.) Decne* is a plant from the Asclepiadaceae subfamily native to Southeast Asia and Thailand, particularly in the northern regions. It is widely used in Thai cuisine and commercial herbal teas. It has been reported to have therapeutic properties in folk medicine, Ayurveda, and homeopathic medicine. *G. inodorum* has been used for centuries to treat diabetes, rheumatoid arthritis, and gout [7]. The many therapeutic phytochemical substances discovered in the leaves of *G. inodorum* include phenolics, flavonoids, terpenoids, triterpenoid saponin, and glycosides. These have been shown to have antioxidant, anti-inflammatory, anti-diabetic, anti-hypoglycemic, anti-adipogenesis, anti-microbial, and anti-cancerous properties [8]. Previous studies of *G. inodorum* leaf extract using in vivo models have found this plant to exhibit potent antimalarial activity and protection against hypoglycemia, dyslipidemia, liver damage, and acute kidney injury, with normalization of hematological parameters that have been dysregulated by *Plasmodium berghei* infection in mice [9–11]. To the best of our knowledge, no studies have examined this plant’s antimalarial activity in combination with artemisinin derivatives as ACT, particularly for the *P. berghei* disease variant. Therefore, this is the first study to investigate the antimalarial effects of combining *G. inodorum* leaf extract with dihydroartemisinin as ACT to treat mice infected with *P. berghei*.

## Methods

### Gymnema inodorum and preparation of extracts

*Gymnema inodorum* leaves were obtained from the Chiangda Organic Company Garden in Chiang Mai, Thailand. The plant was authenticated by a plant biologist at Chiang Mai University, and the voucher specimen (NRU64/036 − 001) was then deposited at Walailak University’s Research Excellence Center for Innovation and Health Products. The plant components were dried in a hot-air oven at 50 °C before being powdered in an electric blender. The dried powdered *G. inodorum* (250 g) was steeped in 750 ml of distilled water at room temperature for seven days, with occasional stirring, to produce a crude aqueous extract. After filtering with Whatman no. 1 filter paper (Whatman International Ltd., Maidstone, UK), the filtrate was collected. Lyophilization was used to obtain a dried powdered form of the aqueous crude extract of *G. inodorum* (GIE) [12]. This was stored at − 20 °C until use. Based on the weight of the animals prior to experimentation, GIE was freshly produced in 20% Tween-80.

### Preparation of standard antimalarial drugs

Dihydroartemisinin (DHA) was obtained from Sigma-Aldrich Co. (St Louis, MO, USA) and stored at − 20 °C. Prior to oral administration, the DHA was dissolved in 20% Tween-80 based on the body weight of the mice (0.1, 1, 5, 10, and 20 mg/kg).

### Experimental animals

Healthy BALB/c male mice weighing 20–25 g at the time of primary infection were obtained from Nomura Siam International Co. Ltd. The mice were kept at a temperature of 22–25 °C with a 12-hour light/12-hour dark cycle. They were fed a commercial pellet diet of 082G with *ad libitum* access to clean tap water.

### Rodent malaria parasite

The ANKA *Plasmodium berghei* strain (PbANKA) was obtained from the Malaria Research and Reference Reagent Resource Center (MR4). This was thawed in a 37 °C water bath prior to use, and 200 µL of the suspension was injected intraperitoneally (IP) into the mice. Parasitemia was assessed daily by microscopic examination of Giemsa-stained blood films, and serial passage was performed when the parasitemia reached 10–20%. Blood was drawn through a cardiac puncture and diluted with normal saline to obtain 1 × 10^7^ parasitized erythrocytes for IP injection.

### Determination of parasitemia

Tail blood from the PbANKA-infected mice was smeared on microscope slides. After air drying, each smeared slide was fixed with absolute methanol and stained with a 10% Giemsa solution for 15 min at room temperature. Parasitic erythrocytes were counted under a light microscope with a 100 × oil immersion lens, and parasitemia was calculated using the formula below.

$$\%\;\mathrm{parasitemia}=\frac{\mathrm{number}\;\mathrm{of}\;\mathrm{parasitized}\;\mathrm{erythrocytes}\;\times100}{\mathrm{total}\;\mathrm{number}\;\mathrm{of}\;\mathrm{erythrocytes}}$$

### Antimalarial assay

The antimalarial investigation began with a standard 4-day suppression test to determine the effective dose (ED_50_) of the drugs (GIE and DHA) [13]. Mice were given IP injections of 1 × 10^7^ parasitized PbANKA erythrocytes (five animals per dosage group). For 4 days, the mice were given GIE (1, 10, 50, 100, and 200 mg/kg) and DHA (0.1, 1, 5, 10, 20 mg/kg) orally via gavage 2 h after infection (days 0–3). The untreated control group received 10 ml/kg of 20%.

Tween-80. On day 4, parasitemia was determined by examining Giemsa-stained blood films under a microscope and calculating the percentage of inhibition using the formula below.

$$\%\mathrm{inhibition}=\frac{\left(\mathrm{parasitemia}\;\mathrm{of}\;\mathrm{the}\;\mathrm{untreated}\;\mathrm{group}-\mathrm{parasitemia}\;\mathrm{of}\;\mathrm{tested}\;\mathrm{group}\right)\;\times\;100}{\mathrm{parasitemia}\;\mathrm{of}\;\mathrm{the}\;\mathrm{untreated}\;\mathrm{group}}$$

### Combination treatment

The ED_50_ (effective dose for 50% of the population) values for both GIE and DHA were used in the combination treatment. The GIE and DHA were combined at fixed ratios of 100/0, 80/20, 60/40, 40/60, 20/80, and 0/100, according to the fixed-ratio approach [14]. Treatment with each ratio combination was tested on the different groups of mice using the traditional 4-day suppression test. On day 4, parasitemia was assessed in the mice, and inhibition percentages were calculated. Points above the joint line indicated synergism, while points around the line or below indicated additive or antagonistic interactions, respectively. A combination index (CI) was created to better understand the interaction of GIE and DHA in their combined effect against PbANKA. The body weights (BWs), packed cell volumes (PCVs), and mean survival time (MST) of the mice in each group were also recorded.

### Determination of body weight and packed cell volume

On day 0 and day 4 post-infection, the BW of each mouse was measured and recorded using a sensitive electronic balance. To estimate PCV, blood was drawn from each mouse’s tail vein to fill 3/4 of the volume of heparinized capillary tubes. The tubes were sealed and spun at 12,000 rpm for 15 min in a microhematocrit centrifuge. The PCV was then calculated using the formula below. On day 0, the PCV was calculated before and after infection. It was then measured again on day 4.

$$\mathrm{PCV}=\frac{\mathrm{volume}\;\mathrm{of}\;\mathrm{packed}\;\mathrm{erythrocytes}\;\times\;100}{\mathrm{total}\;\mathrm{blood}\;\mathrm{volume}}$$

### MST

The mortality of each mouse was tracked and documented throughout the follow-up period, which was from the time of infection until death or a maximum of 30 days survival. For each dosage group and the control group, the MST was calculated using the formula below.

$$\mathrm{MST}=\frac{\mathrm{sum}\;\mathrm{of}\;\mathrm{survival}\;\mathrm{time}\;\mathrm{of}\;\mathrm{mice}\;\mathrm{in}\;\mathrm{the}\;\mathrm{group}}{\mathrm{total}\;\mathrm{number}\;\mathrm{of}\;\mathrm{mice}\;\mathrm{in}\;\mathrm{the}\;\mathrm{group}}$$

### Statistical analysis

GraphPad Prism 6.0 (GraphPad Software, Inc., San Diego, CA, USA) was used to analyze the findings of this investigation. The data were presented as the mean ± standard error of the mean (SEM). A non-linear regression for the sigmoidal dose-response variable slope was used to determine the best-fit ED_50_ value. To compare the means of the control and treatment groups, one-way ANOVAs and Tukey’s post hoc tests were used. The confidence interval was set at 95% and *p* < 0.05 was deemed statistically significant. The CI used to determine synergism (CI < 1), additive effect (CI = 1), and antagonism (CI > 1), was simulated using CompuSyn software (ComboSyn, Inc., USA).

## Results

### PbANKA development in BALB/c mice

**Fig. 1:**
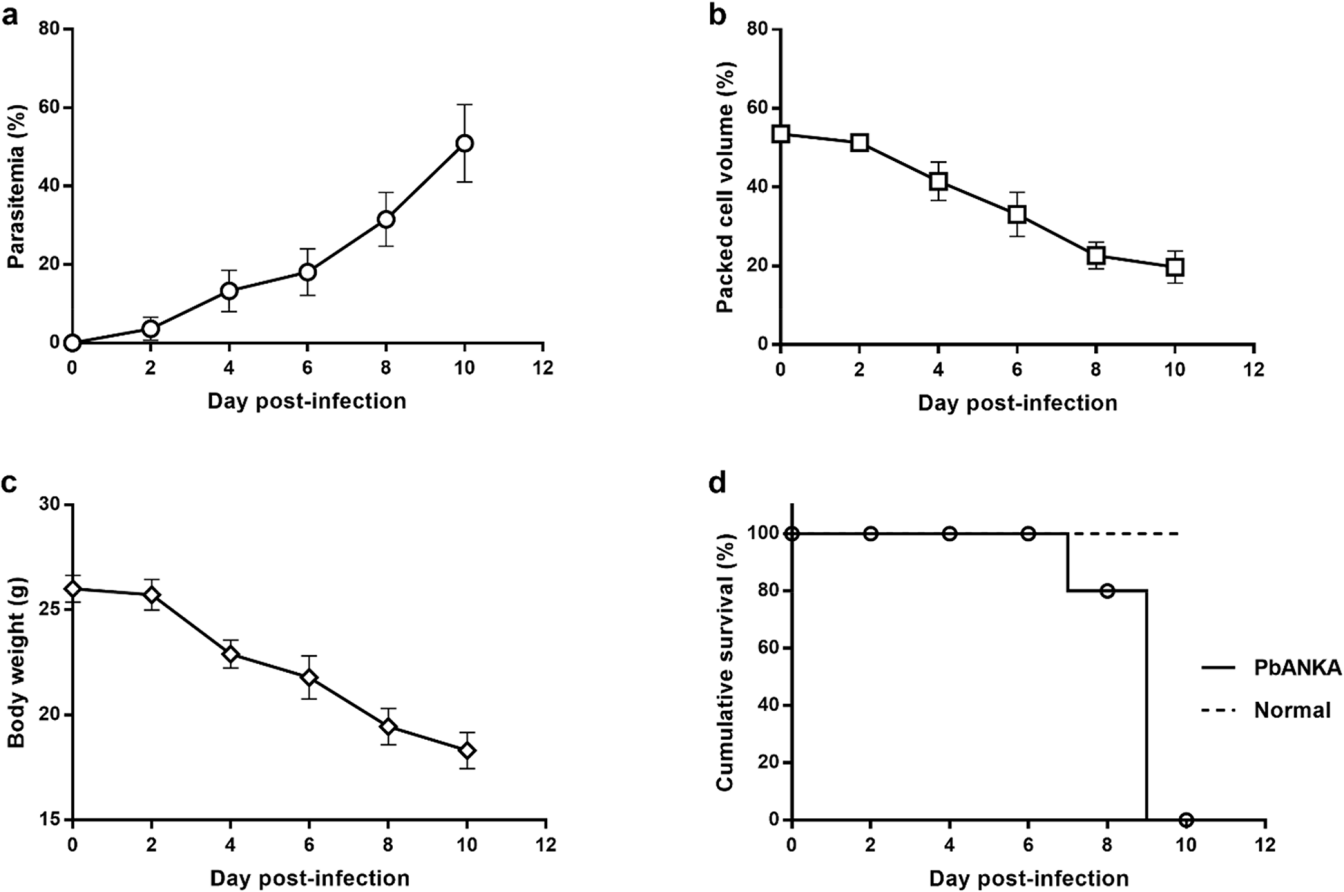
PbANKA infection in BALB/c mice. The mice were infected with 1 × 10^7^ parasitized erythrocytes of PbANKA by IP injection. **a** Parasitemia (%), **b** body weight (g), **c** packed cell volume (%), and **d** mean survival time (days) were monitored. Results are presented as mean ± SEM (*n* = 5)

To investigate the development of PbANKA in mice, parasitemia, BW, PCV, and MST were monitored. As shown in Fig. 1a, parasitemia was first detectable on day 1 post-infection, when it was < 1%. It reached 51% on day 10 post-infection. BW and PCV were markedly decreased (from 26.0 ± 1.45 g to 18.3 ± 1.9 g in BW and from 53.5 ± 1.5% to 19.7 ± 4.1% in PCV, respectively) in infected BALB/c mice (Fig. 1b and c). PbANKA-infected mice died within 10 days post-infection (Fig. 1d).

### Antimalarial activities of DHA and GIE

**Fig. 2:**
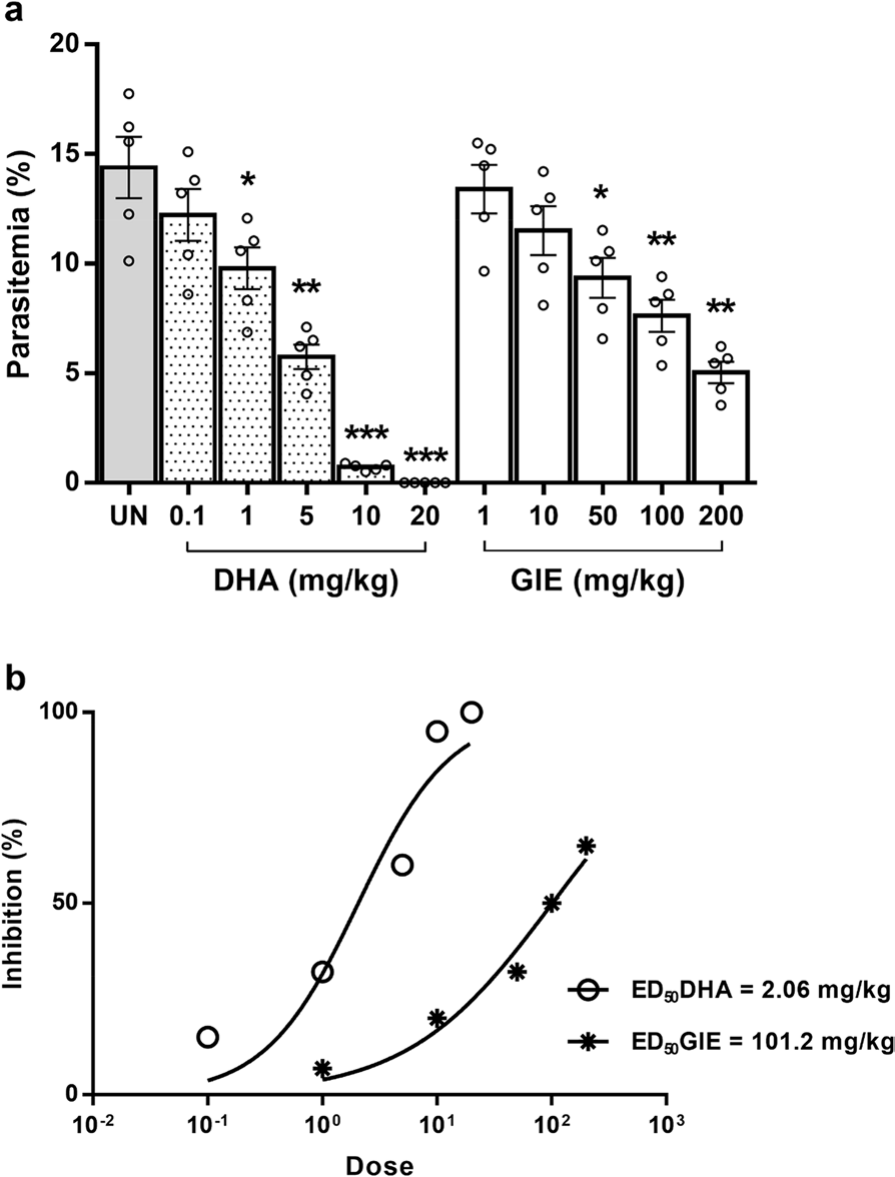
Antimalarial activity of DHA and GIE against PbANKA in infected mice. BALB/c mice were infected with 1 × 10^7^ parasitized erythrocytes of PbANKA by IP injection and subsequently administered DHA (0.1, 1, 5, 10, or 20 mg/kg) and GIE (1, 10, 50, 100, or 200 mg/kg) by gavage for four consecutive days. On day 4, **a** parasitemia (%) was measured and **b** ED_50_ values (mg/kg) were calculated. Results are presented as mean ± SEM (*n* = 5). DHA, dihydroartemisinin; GIE, Gymnema inodorum leaf extract; UN, untreated control. *p < 0.05, **p < 0.01, and ***p < 0.001, compared to untreated control

To investigate the antimalarial activity of DHA and GIE against PbANKA, a standard 4-day suppression test was carried out. As can be seen in Fig. 2a, GIE significantly (*p* < 0.05) inhibited PbANKA in a dose-dependent manner at 50, 100, and 200 mg/kg doses with 32%, 50%, and 65% inhibition, respectively. However, dosages of 1 and 10 mg/kg of GIE showed no antimalarial effect. Moreover, significant (*p* < 0.01) dose-dependent antimalarial activity was also observed in infected mice treated with DHA at doses of 1, 5, 10, and 20 mg/kg with 32%, 60%, 95%, and 100% inhibition, respectively. The ED_50_ values of DHA and GIE against PbANKA were 2.06 (∼2) and 101.2 (∼100) mg/kg, respectively (Fig. 2b).

### Interaction of DHA and GIE against PbANKA-infected mice

**Fig. 3:**
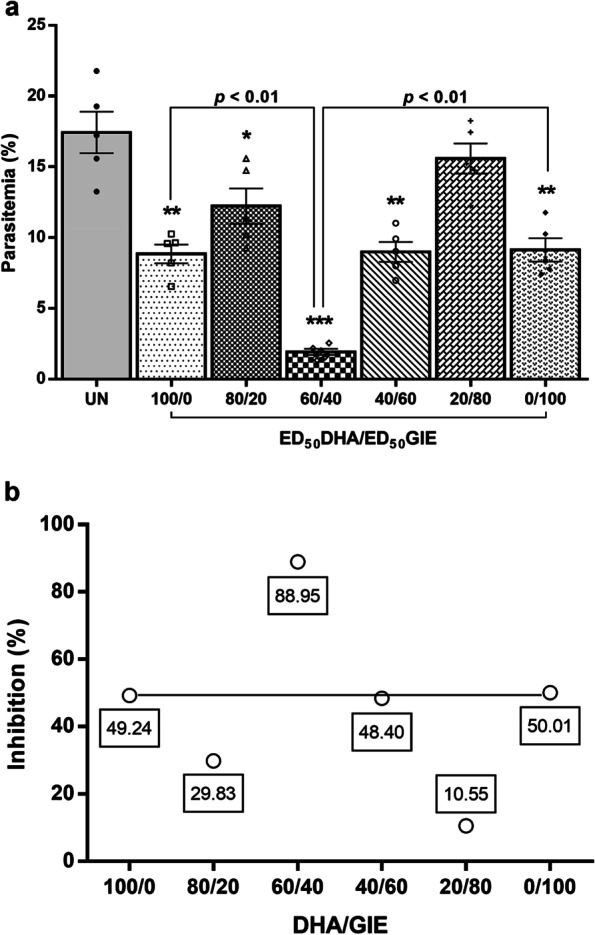
Combined effects of DHA and GIE against PbANKA in infected mice. BALB/c mice were infected with 1 × 10^7^ parasitized erythrocytes of PbANKA by IP injection. They were given combinations between the ED_50_ value of DHA and the ED_50_ value of GIE (100/0, 80/20, 60/40, 40/60, 20/80, or 0/100) by oral gavage for four consecutive days. On day 4, **a** parasitemia (%) was measured, and **b** an interaction line was generated. Results are presented as mean ± SEM (*n* = 5). DHA, dihydroartemisinin; GIE, Gymnema inodorum leaf extract; UN, untreated control. *p < 0.05, **p < 0.01, and ***p < 0.001, compared to untreated control

To investigate the combined effect of DHA/GIE treatment against PbANKA, various dosage combinations were tested. The results of this combination antimalarial treatment for the different dosage ratios are shown in Fig. 3; Table 1. The ratio of 60/40 (DHA/GIE) had the highest significant antimalarial activity (*p* < 0.001) with 88.95% inhibition, which indicated synergistic efficacy (CI = 0.68695). The curve obtained by the other combinations of DHA and GIE and CI values indicated antagonistic interactions.

**Table 1:** Combination index of interaction between DHA and GIE against PbANKA-infected mice

Test		Dose (mg/kg)		CI value
		DHA	GIE	
Combination	100/0	2	0	-
	80/20	1.6	20	1.70625^b^
	60/40	1.2	40	0.68695^a^
	40/60	0.8	60	1.48699^b^
	20/80	0.4	80	3.65127^b^
	0/100	0	100	-

*DHA* dihydroartemisinin, *GIE Gymnema inodorum* extract, *CI* combination inde

^a^ CI < 1; synergism, ^b^ CI > 1; antagonism

### Effects of the interaction of DHA and GIE on BW, PCV, and MST in PbANKA-infected mice

**Fig. 4:**
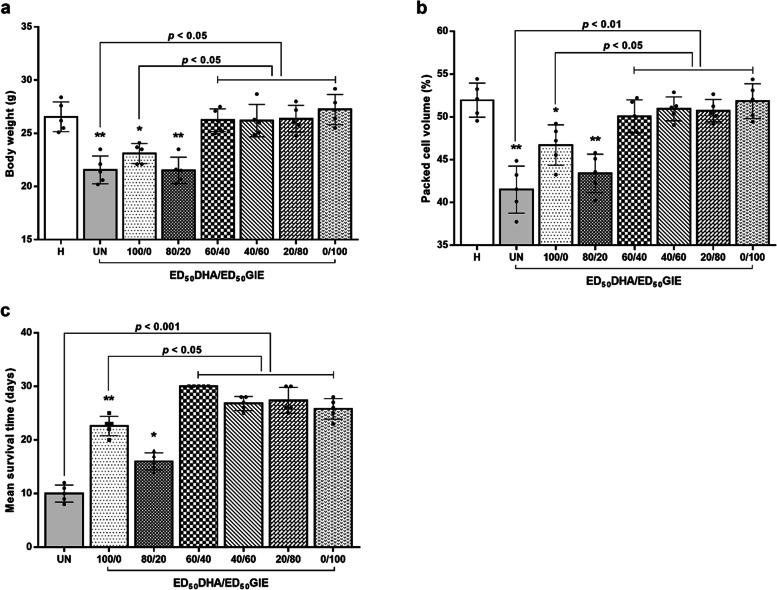
BW, PCV, and MST of PbANKA-infected mice treated with a combination of DHA and GIE. BALB/c mice were infected with 1 × 10^7^ parasitized erythrocytes of PbANKA by IP injection. They were given a combination of DHA and GIE between the ED_50_ values of the two (DHA/GIE dosage ratios of 100/0, 80/20, 60/40, 40/60, 20/80, or 0/100) by oral gavage for four consecutive days. On day 4, **a** BW (g), **b** PCV (%), and **c** MST (day) were measured. Results are presented as mean ± SEM (*n* = 5). DHA, dihydroartemisinin; GIE, Gymnema inodorum leaf extract; H, healthy control; UN, untreated control. *p < 0.05 and **p < 0.01 compared to healthy control

To investigate the effects of the DHA/GIE combination treatment on complications resulting from PbANKA infection, the BW, PCV, and MST were measured in each dosage group. PbANKA infection significantly decreased BW and PCV, compared to a healthy control group (*p* < 0.01) (Fig. 4a and b). As expected, the infected mice treated with a DHA/GIE ratio of 60/40 were significantly protected from BW loss and PCV reduction (*p* < 0.05), compared to untreated mice and mice treated with DHA alone. Interestingly, the ratios of 40/60, 20/80, and 0/100 that increased the proportion of GIE also showed significant (*p* < 0.05) protective effects on BW loss and PCV reduction. Additionally, the MST of the 60/40 dosage group was significantly longer than that of the other groups (*p* < 0.05) (Fig. 4c). The 40/60, 20/80, and 0/100 dosages all significantly prolonged MST, compared with untreated mice and those treated with DHA alone (*p* < 0.05).

## Discussion

With antimalarial medication resistance on the rise, new therapeutic options are critical. New medications, particularly artemisinin-based combinations, must be used alongside traditional antimalarial agents [4]. In this study, we evaluated the combined antimalarial effects of GIE, an indigenous medicinal plant and functional food in Thailand, and DHA on mice infected with PbANKA. Mice given 50, 100, or 200 mg/kg GIE showed significant disease inhibition of 32, 50, and 65%, respectively, compared to untreated control mice. Plant extracts with in vivo antimalarial activity greater than 30% inhibition are considered to have active effects [15]. Thus, GIE can be classified as an active antimalarial. This is consistent with previous research [10]. GIE’s antimalarial activity could be attributable to the action of one of its bioactive metabolites or the combined action of several of them. These include phenols, flavones, alkaloids, anthraquinones, quinones, tannins, and triterpene saponins [7, 16, 17]. Gymnemic acids, the major active compounds in GIE, are likely to play an important role in its antimalarial activity [18–20]. The specific mechanism(s) of action could be antioxidant activity, intercalation with parasite DNA, suppression of parasite fatty acid and protein production, increased erythrocyte oxidation, immunomodulation, parasite invasion inhibition, or other undiscovered mechanisms [21, 22].

We tested GIE combined with DHA as an antimalarial treatment in mice infected with PbANKA. Combination DHA/GIE treatment at a dosage ratio of 60/40 was found to be the most effective, with 88.95% inhibition, which was indicative of a synergistic effect. In combination with DHA, GIE demonstrated significant antimalarial activity when compared to GIE or DHA monotherapies. DHA’s mechanisms of action have been hypothesized to include the formation of free radicals or reactive metabolites, as well as the blocking of parasite nutrition through interference with its passage through cellular membranes [23–25]. Furthermore, DHA inhibits parasite SERCA (sarco/endoplasmic reticulum Ca^2+^-ATPase) and is a good target for artemisinin and its derivatives [26]. GIE has been linked to several metabolic pathways, and its combination with DHA produced the best synergy and most positive results. However, more research is needed before any firm conclusions can be drawn.

A loss of BW is a known symptom of malaria infection in mice, and changes in BW are often used as a metric to assess the antimalarial efficacy of treatments [27]. Compared to the untreated control, PbANKA-infected mice given a DHA/GIE combination at the dose ratios of 60/40, 40/60, 20/80, and 0/100 showed significant increases in BW. A lack of similar outcomes from DHA monotherapy demonstrated that GIE can prevent malaria-related loss of BW. This could be because GIE contains substances that influence hunger [28]. GIE activity in the 80/20 combination ratio was insufficient to significantly reduce BW loss.

PCV reduction during malaria infection was also evaluated in this study. The PCV of the untreated control group was reduced as a result of PbANKA infection, causing rapid hemolysis [29, 30]. The absence of significant PCV loss in DHA/GIE combination-treated animals at dosage ratios of 60/40, 40/60, 20/80, and 0/100 suggests that GIE protects against PCV loss during malaria infection. This may be due to the activation of erythropoietin, which facilitates the production of new erythrocytes in the bone marrow. This finding supports previous research that found GIE to protect against decreases in PCV in rodent malaria [11].

MST is another parameter used to assess the antimalarial activity of plant extracts. In this study, all DHA/GIE doses significantly prolonged MST compared with the untreated control, especially at the 60/40 ratio. This adds to the existing evidence that inhibiting PbANKA reduces the total parasitic infection in experimental mice. *G. inodorum* can prolong MST in infected mice [31]. When combined with DHA, GIE appears to have synergistic antimalarial effects on PbANKA.

## Conclusion

This study is the first to provide research evidence for the antimalarial activity of combined GIE and DHA in PbANKA-infected mice. The combination of DHA and GIE was also found to protect against loss of body weight and packed cell volume reduction caused by prolonged malaria infection. Based on our results, we recommend GIE as an alternative antimalarial ingredient for future use with traditional antimalarial medications such as DHA. However, more research on the mechanisms of action of GIE and its combination with DHA in malaria treatment is required.

## Data Availability

The data that support the findings of this study are openly available on Figshare at https://figshare.com/s/4c16cf376cbabd67e740 (DOI: 10.6084/m9.figshare.19358474). A preprint has previously been published [30].

## Abbreviations

ACT: Artemisinin-based combination therapies
BW: Body weights
CI: Combination index
MST: Mean survival time
PCV: Packed cell volumes
SEM: Standard error of the mean
